# Relationship between TP53 and interleukin-6 gene variants and the risk of types 1 and 2 diabetes mellitus development in the Kermanshah province

**DOI:** 10.25122/jml-2019-0150

**Published:** 2021

**Authors:** Lida Haghnazari, Ramin Sabzi

**Affiliations:** 1. Department of Clinical Biochemistry, School of Medicine, Kermanshah University of Medical Sciences, Kermanshah, Iran; 2. School of Medicine, Kermanshah University of Medical Sciences, Kermanshah, Iran

**Keywords:** type 1 diabetes mellitus, type 2 diabetes mellitus, TP53, interleukin-6, single nucleotide polymorphism

## Abstract

Diabetes mellitus (DM) is a metabolic disorder that results from insufficient secretion or insulin resistance, or both. Insulin secretion deficiency leads to chronic hyperglycemia along with impaired metabolism of proteins, lipids, and carbohydrates. This study aimed to investigate the TP53 gene SNP (single nucleotide polymorphism) rs1042522 genotype and the interleukin-6 (IL-6) gene SNP rs1800795 genotype in DM and control groups. This study was performed on 70 patients with type 1 DM, 100 patients with type 2 DM without related complications, 66 control subjects for type 1 DM, and 95 control subjects for type 2 DM. The control groups were matched regarding age and gender and did not have a familial relationship with the patient groups. All the subjects were residents of Kermanshah, located in the western part of Iran. Polymorphisms of TP53 and IL-6 genes were determined by the polymerase chain reaction-restriction fragment length polymorphism (PCR-RFLP) method. Lipid profile, fasting blood glucose, and HbA1c were measured using the ELISA and immunoturbidometric methods. The frequency of genotypes (CC, CG, GG) of the TP53 gene codon 72 in type 1 DM and its control group were significantly different (P= 0.013). Likewise, the frequency of genotypes (CC, CG, GG) of the TP53 gene codon 72 was significantly different between type 2 DM and control groups (P <0.001). The frequency of genotypes (GG, GC, CC) of G174C polymorphisms in the IL-6 gene was different between type 1 DM and control group as well as between type 2 DM and its control group, but it was not statistically significant. SNP rs1042522 genotypes in the dominant form (CG + GG vs. CC) (OR= 3.880; P < 0.001) and alleles G vs. C alleles (OR= 0.384; P < 0.001) increased the risk of type 2 DM significantly. There was no significant difference between type 1 and type 2 DM groups and respected control groups regarding the frequency of the IL-6 gene SNP rs1800795 alleles. The G allele of SNP rs1042522 encoding the TP53 gene increases the risk of developing DM in the population of the Kermanshah province, Iran.

## Introduction

Diabetes mellitus (DM) is one of the most common metabolic diseases caused by chronic hyperglycemia associated with abnormalities of the metabolism of lipids, glucose, and proteins. It can lead to defects in the secretion of insulin, function of insulin, or both. Its prevalence is increasing worldwide [1]. Today, DM classification is done on the ground of the pathogenetic pathways that lead to hyperglycemia. In this classification, two large DM groups have been defined, namely, type 1 DM and type 2 DM [2]. DM is a chronic disease associated with cardiovascular diseases, obesity, cancer, and respiratory diseases [3].

The prevalence of type 1 DM has a wide range across various geographical locations. For example, there is evidence that the prevalence of DM is 35 times higher in Finland than in Japan [4]. The highest incidence of type 2 DM has been reported in Scandinavian countries (between 17.6% and 28.6%) [5]. The highest prevalence of this disease is reported in India, China, and the United States [6]. According to the World Health Organization (WHO) reports, the prevalence rates of type 2 DM in Iran in 1995, 2000, and 2025 were 5.5%, 5.7%, and 6.8%, respectively [7].

In type 1 DM, the pancreas does not secrete enough insulin. Although this process usually starts before the age of 30, the process of autoimmune destruction of beta cells of the pancreas can occur at any age [2, 8]. Type 2 DM is characterized by varying insulin resistance levels, insulin secretion deficiency, and increased glucose production. Usually, before DM develops, periods of impaired fasting glucose (IFG) or impaired glucose tolerance (IGT) are seen [2]. This type of DM is the most common type consisting of 90% of all DM patients [9].

One of the proteins with tumor suppression properties is the p53 protein. Recently, the relationship between p53 and human body metabolism has become a new goal in studies [10]. P53 and its family members directly affect various pathways of metabolism and enable cells to respond to metabolic stress. It has been shown that p53 regulates glycolysis negatively [11]. In humans, P53 is encoded by the TP53 gene on the small arm of chromosome 17. The length of the gene is 20,000 base pairs (bp). Its first exon is non-coding, and the first intron is also very long (10,000 bp). The coding sequence consists of 5 regions, of which exons 2, 5, 6, 7, and 8 of this gene are strongly protected among vertebrate species [12, 13]. If the TP53 gene is damaged, suppression of tumors will be greatly reduced. Individuals who inherit only a healthy and active TP53 gene are highly likely to develop malignancy early in the pubertal period. 

Pro-inflammatory cytokines such as interleukin-6 (IL-6) have an independent relationship with cardiovascular risk factors, including high blood pressure, reduced high-density lipoprotein cholesterol (HDL-C) levels, and increased body mass index (BMI) [14]. Interleukin 6 (IL-6) is a multi-purpose cytokine expressed in many tissues involved in regulating energy balance, such as adipose tissue, skeletal muscle, and hypothalamus. High levels of cytokines, including IL-6, are risk factors for developing type 2 DM [15].

Given the importance and evidence presented, it is clear that p53 plays a key role in DM and its severity. In most cases, the activation of p53 leads to more severe DM. For example, this protein is induced and is active in many animal and human cells with DM, and in most cases, the inhibition of p53 decreases the severity of diabetes. On the other hand, the C-174C genotype of the IL-6 gene is associated with insulin resistance. However, the role of the variants of TP53 and IL-6 has not been clarified clearly in DM. There is not enough evidence regarding the role of SNP (single nucleotide polymorphism) rs1042522 in the TP53 gene and SNP rs1800795 gene in the IL-6 gene in the development of type 1 and type 2 DM in the western part of Iran. Therefore, we decided to investigate the role of these variants in the development of type 1 and type 2 DM in this area. 

## Material and Methods

The study population of this cross-sectional study consisted of patients with types 1 and 2 DM who were residents of the Kermanshah province, located in the western part of Iran. 

Based on the results of previous studies and the relationship between SNP rs1042522 polymorphisms in the TP53 and SNP rs1800795 gene in the IL-6 gene with diabetes, and considering the frequency of mutation genotypes for each genotype with a 95% confidence interval and a power of 90%, the required sample size was calculated as 170 patients (70 patients with type 1 DM and 100 patients with type 2 DM). In addition to the DM groups, two control groups (one for type 1 DM - 66 subjects and one group for type 2 DM - 95 subjects) were considered. The subjects of control groups were recruited from residents of Kermanshah. The control subjects were matched based on gender and age to the respective DM groups. 

The samples that were used in this study were extracted from previously approved studies (registration numbers 94254 and 94094) at the Diabetes Center of the Kermanshah University of Medical Sciences and were located at the Taleghani Hospital and Mehr Pathobiology Laboratory. 

Informed consent was obtained from the patients. DNA extraction from blood samples of the subjects was done and collected in ethylenediaminetetraacetic acid-containing tubes by the phenol-chloroform method. The concentration of the extracted DNA was measured using the NanoDrop ™ spectrophotometer system.

### Statistical analysis

Descriptive indices, including frequency and percentage, were used to describe categorical variables. The Chi-squared test was used to compare frequency distribution between DM and control groups. Continuous variables are presented by mean (standard deviation), and a comparison of these variables between the groups was made using independent samples t-test. 

## Results

A total of 331 subjects were included for the final analysis. The frequency distribution of the included patients in four studied groups and demographic characteristics of the subjects studied are presented in Table 1.

**Table 1.** Demographic (age and gender) characteristics of the patients in four studied groups.

**Group**	**No.**	**Age**	**Gender**	
			**Male**	**Female**
Type 1 DM	70	19.65 (±7.33)	35	35
Control for type 1 DM	66	18.14 (±8.34)	34	32
**P-value**		**NS**	**NS**	
Type 2 DM	100	56.18 (±8.94)	57	43
Control for type 2 DM	95	53.84 (±9.14)	51	44
**P-value**		**NS**	**NS**	

NS – non-significant.

Table 2 presents a comparison of BMI, waist circumference, HbA1c, fasting blood glucose (FBG), urea, creatinine, and lipid profiles between the study groups. In type 1 DM and its control groups, a statistically significant difference was observed regarding the levels of HBA1C, fasting blood glucose (FBG), LDL-C, and HDL-C between type 1 DM and its control group. However, no difference was seen regarding other variables. In type 2 DM and its control groups, HBA1C, fasting blood glucose (FBG), and HDL-C were different.

**Table 2.** Comparison of BMI, waist circumference, HbA1c, fasting blood glucose, urea, creatinine, and lipid levels between the study groups.

**Variables**	**Type 1 DM**	**Control for type1 DM**	**Sig.**	**Type 2 DM**	**Control for type 2 DM**	**Sig.**
**BMI, kg/m**^**2**^	21.59 (±4.11)	22.05 (±3.11)	0.84	27.48 (±3.86)	26.82 (±2.16)	0.54
**Waist circumference, cm**	76.03 (±11.84)	76.70 (±11.31)	0.98	101.45 (±11.35)	102.16 (±8.30)	0.96
**HBA1C, %**	8.51 (±1.39)	4.71 (±0.3)	<0.001	8.16 (±1.41)	4.8 (±0.36)	<0.001
**FBG, mg/dL**	180.16 (±78.96)	90.79 (±6.59)	<0.001	192.42 (±66.58)	94.27 (±6.73)	<0.001
**TC, mg/dL**	166.44 (±29.64)	174.17 (±30.54)	0.55	176.66 (±45.80)	190.35 (±33.29)	0.042
**Triglyceride, mg/dL**	112 (±51.56)	127.73 (±55.62)	0.43	136 (±82.72)	154.11 (±58.26)	0.2
**LDL-C, mg/dL**	86 (78-94.5)	100 (90-115)	<0.001	109 (97-192)	101 (87.3-118)	0.08
**HDL-C, mg/dL**	49 (43-54)	42.5 (38.8-48)	<0.001	40 (34.3-45)	44 (37-50)	0.003
**BUN, mg/dL**	27.65 (±6.25)	25.30 (±5.17)	0.12	29.82 (±7.02)	30.35 (±7)	0.94
**Creatinine, mg/dL**	0.8 (±0.12)	0.79 (±0.12)	0.91	0.89 (±0.15)	0.9 (±0.17)	0.9

DM – diabetes mellitus; BMI – body mass index; FBG – fasting blood glucose; TC – total cholesterol; LDL-C – low-density lipoprotein cholesterol; HDL-C – high-density lipoprotein cholesterol; BUN – blood urea nitrogen.

The frequency occurrence of genotypes (CC, CG, GG) of the TP53 gene SNP rs1042522 in the studied groups is presented in Table 3. As observed, the frequency of alleles (CC, CG, and GG) was different between type 1 DM and its control group as well as between type 2 DM and its control group.

**Table 3.** Comparison of the frequency occurrence of genotypes (CC, CG, GG) of the TP53 gene SNP rs1042522 in the studied groups.

**SNP rs1042522**	**Type 1 DM**	**Control for type1 DM**	**Sig.**	**Type 2 DM**	**Control for type 2 DM**	**Sig.**
**CC**	22 (31%)	31 (47%)	0.013	23 (23%)	51 (53.7%)	<0.001
**CG**	38 (53.5%)	19 (28.8%)		46 (46%)	29 (30.5%)	
**GG**	11 (15.5%)	16 (24.2%)		31 (31%)	15 (15.8%)	
**CC**	22 (31%)	31 (47%)	0.055	23 (23%)	51 (53.7%)	<0.001
**CG + GG**	49 (69%)	35 (53%)		77 (77%)	44 (44.3%)	
**C allele**	82 (57.7%)	81 (61.4%)	0.54	92 (46%)	131 (68.9%)	<0.001
**G allele**	60 (42.3%)	51 (38.6%)		108 (54%)	59 (31.1%)	

The results showed that SNP rs1042522 genotypes in the dominant form (CG + GG vs. CC) (OR= 1.973; P = 0.056) and G vs. C alleles (OR= 0.860; P = 0.542) increased the risk of type 1 DM, but were not statistically significant. In addition, SNP rs1042522 genotypes in the dominant form (CG + GG vs. CC) (OR= 3.880; P < 0.001) and G vs. C alleles (OR= 0.384; P < 0.001) increased the risk of type 2 DM significantly.

The frequency occurrence of genotypes (CC, CG, GG) of the IL-6 gene SNP rs1800795 in the studied groups is presented in Table 4. As observed, there was no significant difference between type 1 and type 2 DM groups and respected control groups regarding the frequency of the occurrence of the IL-6 gene SNP rs1800795 alleles.

**Table 4.** Comparison of the frequency occurrence of genotypes (CC, CG, GG) of the IL-6 gene SNP rs1800795 in the studied groups.

**SNP rs1800795**	**Type 1 DM**	**Control for type1 DM**	**Sig.**	**Type 2 DM**	**Control for type 2 DM**	**Sig.**
**GG**	42 (59.2%)	44 (67.7%)	0.58	48 (48%)	50 (52.6%)	0.763
**GC**	25 (35.2%)	20 (30.3%)		45 (45%)	40 (42.1%)	
**CC**	4 (5.6%)	2 (3%)		7 (7%)	5 (5.3%)	
**GG**	42 (59.2%)	44 (66.7%)	0.36	48 (48%)	50 (52.6%)	0.518
**GC + CC**	29 (40.8%)	22 (33.3%)		52 (52%)	45 (47.4%)	
**G allele**	109 (76.8%)	108 (81.8%)	0.303	141 (70.5%)	140 (73.7%)	0.484
**C allele**	33 (23.2%)	24 (18.2%)		59 (29.5%)	50 (26.3%)	

The results showed that SNP rs1800795 genotypes in the dominant form (GC + CC vs. GG) (OR= 1.381; P = 0.364) and C vs. G alleles (OR= 0.734; P = 0.304) increased the risk of type 1 DM, but were not statistically significant. In addition, SNP rs1800795 genotypes in the dominant form (GC + CC vs. GG) (OR= 1.204; P= 0.518) and C vs. G alleles (OR= 0.854; P= 0.484) increased the risk of type 2 DM, but this was not statistically significant.

Tables 5 and 6 present a comparison of the studied variables in type 1 and type 2 DM groups and related control groups based on SNP rs1042522 and SNP rs1800795 genotypes of the TP53 and IL-6 genes.

**Table 5.** Comparison of the studied variables between type 1 diabetes mellitus and its control group according to SNP rs1042522 and SNP rs1800795 genotypes of the TP53 and IL-6 genes.

**Variables**	**CC genotype SNP rs1042522**			**CG + GG genotype SNP rs1042522**			**GG genotype SNP rs1800795**			**GC + CC genotype SNP rs1800795**		
	**DM**	**Control**	**Sig.**	**DM**	**Control**	**Sig.**	**DM**	**Control**	**Sig.**	**DM**	**Control**	**Sig.**
**BMI, kg/m2**	21.96 (3.82)	22.08 (2.73)	<0.001	26.49 (3.29)	26.54 (1.85)	0.067	21.56 (4.39)	22.39 (3.45)	<0.001	27.77 (4.25)	27.11 (2.12)	0.011
**HBA1C, %**	8.52 (1.49)	4.75 (0.34)	<0.001	8.21 (1.36)	4.89 (0.32)	<0.001	8.77 (1.39)	4.76 (0.32)	<0.001	8.33 (1.32)	4.80 (0.41)	<0.001
**FBG, mg/dL**	174.27 (87.82)	91.26 (6.38)	<0.001	191.43 (69.96)	95.27 (5.52)	<0.001	189.88 (99.59)	90 (6.53)	<0.001	195.94 (72.19)	93.24 (6.19)	<0.001
**TC, mg/dL**	167.82 (36.96)	176.29 (30.35)	0.048	179 (45.87)	191.63 (32.85)	0.048	166.40 (31.94)	177.64 (31.06)	0.098	181.67 (47.97)	184.84 (32.55)	0.098
**Triglyceride, mg/dL**	121.91 (72.10)	127.45 (46.79)	0.015	125.91 (68.62)	151.24 (61.30)	0.15	107.29 (55.18)	132.80 (59.36)	0.003	131.23 (66.03)	154.64 (58)	0.003
**LDL-C, mg/dL**	87.23 (12.48)	103 (14.77)	<0.001	103.70 (22.10)	107.49 (15.23)	<0.001	86.57 (15.39)	102.39 (13.87)	<0.001	104.44 (24.76)	105.56 (16.24)	<0.001
**HDL-C, mg/dL**	50.18 (8.22)	44.48 (5.37)	0.002	41.57 (7.74)	44.14 (8.35)	0.002	48.40 (7.71)	43.18 (6.24)	<0.001	41.15 (7.91)	42.34 (7.82)	<0.001
**BUN, mg/dL**	31 (6.19)	26.48 (5.75)	0.025	29.09 (7.07)	30.94 (7.35)	0.025	27.29 (5.68)	25.77 (5.34)	<0.001	31.31 (8.22)	29.70 (5.02)	<0.001
**Creatinine, mg/dL**	0.86 (0.09)	0.81 (0.13)	0.018	0.86 (0.15)	0.92 (0.18)	0.018	0.8 (0.11)	0.8 (0.15)	0.014	0.92 (0.16)	0.88 (0.12)	<0.001

DM – diabetes mellitus; BMI – body mass index; FBG – fasting blood glucose; TC – total cholesterol; LDL-C – low-density lipoprotein cholesterol; HDL-C – high-density lipoprotein cholesterol; BUN – blood urea nitrogen.

**Table 6.** Comparison of the studied variables between type 2 diabetes mellitus and its control group according to SNP rs1042522 and SNP rs1800795 genotypes of the TP53 and IL-6 genes.

**Variables**	**CC genotype SNP rs1042522**			**CG + GG genotype SNP rs1042522**			**GG genotype SNP rs1800795**			**GC + CC genotype SNP rs1800795**		
	**DM**	**Control**	**Sig.**	**DM**	**Control**	**Sig.**	**DM**	**Control**	**Sig.**	**DM**	**Control**	**Sig.**
**BMI, kg/m2**	21.22 (4.30)	22.03 (3.45)	<0.001	27.78 (3.99)	27.05 (2.48)	<0.001	21.29 (3.82)	21.38 (2.25)	0.011	27.21 (3.5)	26.52 (2.19)	<0.001
**HBA1C, %**	8.70 (1.39)	4.68 (0.27)	<0.001	8.15 (1.43)	4.84 (0.42)	<0.001	8.47 (1.46)	4.63 (0.27)	<0.001	8.01 (1.5)	4.94 (0.29)	<0.001
**FBG, mg/dL**	181.67 (86.17)	90.37 (6.84)	<0.001	192.72 (66.02)	93.11 (7.81)	<0.001	164.17 (60.11)	92.36 (6.59)	<0.001	189.18 (61.50)	95.42 (7.18)	<0.001
**TC, mg/dL**	169.78 (25.78)	172.29 (31.03)	0.076	175.96 (46.06)	188.86 (34.11)	0.076	173.17 (25.38)	167.23 (28.91)	0.002	172.04 (43.67)	196.47 (33.39)	0.002
**Triglyceride, mg/dL**	101.10 (40.14)	127.97 (63.11)	<0.001	139.01 (86.67)	157.43 (55.04)	<0.001	107.93 (49.37)	117.59 (46.89)	0.037	140.40 (96.05)	153.51 (59.19)	0.037
**LDL-C, mg/dL**	84.04 (16.15)	99.09 (15.44)	<0.001	101.53 (25.39)	105.80 (17.93)	<0.001	82.66 (14.59)	98 (17.38)	<0.001	99.81 (24.44)	107.98 (16.81)	<0.001
**HDL-C, mg/dL**	50.86 (7.74)	42.11 (7)	<0.001	40.01 (7.62)	44.77 (10.22)	<0.001	53.90 (6.93)	43.32 (6.71)	<0.001	99.81 (24.44)	46.76 (10.14)	<0.001
**BUN, mg/dL**	26.20 (6)	24.26 (4.43)	<0.001	30.04 (7.05)	29.66 (6.59)	<0.001	28.28 (7.44)	24.36 (4.81)	0.004	28.44 (5.44)	31.07 (8.7)	0.097
**Creatinine, mg/dL**	0.78 (0.11)	0.79 (0.13)	<0.001	0.9 (0.16)	0.88 (0.17)	<0.001	0.82 (0.12)	0.77 (0.09)	0.004	0.87 (0.15)	0.92 (0.23)	0.069

DM – diabetes mellitus; BMI – body mass index; FBG – fasting blood glucose; TC – total cholesterol; LDL-C – low-density lipoprotein cholesterol; HDL-C= high-density lipoprotein cholesterol; BUN= blood urea nitrogen.

## Discussion

The aim of this study was to determine the role of SNP rs1042522 variants of the TP53 gene and SNP rs1800795 variants of the IL-6 gene in types 1 and 2 DM in western Iran. The obtained results showed that the frequency of the GG genotype of SNP rs1042522 genotype was more prevalent in DM groups than in control groups. Also, the frequency of CG + GG genotype was higher among DM patients compared to control subjects. Furthermore, the frequency of the G allele was higher in DM groups than in control groups. In the present study, it was found that individuals carrying at least one copy of the G (CG or GG) allele had a risk of 0.86 to develop type 1 DM in comparison to the CC genotype. The CG + GG genotype increased the risk of type 1 DM by 1.973 times compared to the CC genotype. There were significant differences regarding genotype frequency between DM and control groups, but no significant difference was seen regarding the risk and SNP rs1042522 allele between the DM and control groups.

A previous study showed that a significant difference existed regarding the distribution of SNP rs1042522 genotypes between type 1 DM and control group in 281 children with type 1 DM and 730 healthy subjects in the Italian population. The GG genotype seems to increase the risk of type 1 DM, while the CC genotype protects against it [16]. The results of this study were consistent with the findings of the present study. Also, another study determined the association of p53 codon 72 polymorphism with the probability of developing type 1 DM in Russia. Those with the G allele had a higher risk of developing type 1 DM. However, no meaningful relationship was found between the patient group and the control group [17], inconsistent with the current results.

In the current study, the frequency distribution of the GG genotype from SNP rs1042522 was higher in the patient group compared to the control group. Also, the frequency of the CG + GG genotype in the DM group was higher compared to the control group. 

In a former study conducted on 273 people with type 2 DM and 237 healthy people in China, it was noted that the frequency distribution of SNP rs1042522 genotypes was significantly different between patients with type 2 DM and the control group. It was stated that the G allele is likely to increase the apoptosis of pancreatic beta cells and insulin secretion [18]. The results of this study are consistent with our findings. Similarly, a recent study found that there was a significant difference in the distribution of SNP rs1042522 genotypes between patients with type 2 DM and the control group. In this study, as in a study conducted in China, the G allele increases the apoptosis of beta cells in the pancreas, in agreement with our results [19]. 

Che-Pei Kung *et al.* showed that rats with the GG genotype (arginine) had less resistance to weight gain than those with the CC (proline) genotypes, which resulted in increased insulin resistance and type 2 diabetes development [20]. In a study of 69 diabetic patients and 147 controls, Gloria *et al.* concluded that the genotype GG (arginine) SNP rs1042522 of the P53 gene is a potent inducer of apoptosis. The increase in p53 in the adipose tissue causes insulin resistance. However, there was no significant difference between diabetes patients and the control group, which contradicts the current findings [21].

In a former study conducted on 235 people with type 2 DM and 255 healthy subjects in the Italian population, it was shown that people with the C (GC + CC) allele had higher resistance to insulin compared to those who have the G (GG) allele. However, there was no significant difference between the DM group and the control group [22]. The results of this study are consistent with the findings of the present study. In another study including 242 obese people in Iran, it was stated that in obese patients with the C (GC + CC) allele, fasting blood glucose was significantly higher than those with the GG genotype. The authors concluded that the C allele is not likely to affect the weight gain of the Iranian population [23], which is relatively compatible with our findings.

Ghavimi and colleagues concluded that the frequency distribution of SNP rs1800795 genotypes of IL-6 gene between patients and control group was not significant in 120 people with diabetes and 120 healthy subjects from Isfahan, Iran. They concluded that this SNP does not cause diabetes in the population of Isfahan [1]. In a review article on 5601 people with type 2 DM and 17,019 healthy subjects, there was evidence that the G174C polymorphism of the IL-6 gene was associated with type 2 DM, and those with the C allele were 9% more likely to develop diabetes than those with the GG genotype [24]. These observations contradict the current findings. Saxena *et al.* found that those with homozygous C (CC) genotype showed the highest susceptibility to DM development in 213 diabetic patients and 147 healthy subjects among North Indian populations. Also, the frequency distribution of SNP rs1800795 genotypes of the IL-6 gene was significantly different between the patients and the control group [25], which is in contrast with the findings that we observed.

## Conclusion

Our findings showed that the SNP rs1042522 variant of the TP53 encoding gene was significantly different between DM and control groups. Patients with type 1 and 2 DM who are carriers of the G allele from SNP rs1042522 encode TP53 had a higher risk of DM. The SNP rs1800795 variant of the IL-6 encoding gene differed in the control group of type 1 and type 2 patients but did not differ significantly. Patients with type 1 and type 2 DM of SNP rs1800795, an IL6-encoding gene, increase the risk of DM. Due to the lack of association between IL-6 174G/C in this study, it is suggested that other polymorphisms of this gene are also examined, and studies with larger sample sizes should be conducted. Meanwhile, the measurement of serum levels of the TP53 and IL-6 gene can be investigated to determine their association with DM.

## Acknowledgments

This article is based on a thesis submitted to the graduate office in the partial fulfillment of the requirements for the degree of MSc in Clinical Biochemistry by Ramin Sabzi at the Kermanshah University of Medical Sciences, Faculty of Medicine. Financial support was given by the Research Council of the Kermanshah University of Medical Sciences (Grant number: 96038).

## Ethical approval

The approval for this study was obtained from the Ethics Committee of the Kermanshah University of Medical Sciences (Approval ID: ir.kums.rec1395.790).

## Conflict of interest

The authors declare that there is no conflict of interest.
